# In-Law Relationships in Evolutionary Perspective: The Good, the Bad, and the Ugly

**DOI:** 10.3389/fsoc.2021.683501

**Published:** 2021-06-04

**Authors:** Martin Daly, Gretchen Perry

**Affiliations:** ^1^ Department of Psychology, Neuroscience and Behaviour, McMaster University, Hamilton, ON, Canada; ^2^ School of Social Work, University of Canterbury, Christchurch, New Zealand

**Keywords:** affines, cooperation and conflict, daughter-in-law, in-laws, kinship, mother-in-law, spousal conflict

## Abstract

In-laws (relatives by marriage) are true kin because the descendants that they have in common make them “vehicles” of one another’s inclusive fitness. From this shared interest flows cooperation and mutual valuation: the good side of in-law relationships. But there is also a bad side. Recent theoretical models err when they equate the inclusive fitness value of corresponding pairs of genetic and affinal (marital) relatives-brother and brother-in-law, daughter and daughter-in-law-partly because a genetic relative’s reproduction always replicates ego’s genes whereas reproduction by an affine may not, and partly because of distinct avenues for nepotism. Close genetic relatives compete, often fiercely, over familial property, but the main issues in conflict among marital relatives are different and diverse: fidelity and paternity, divorce and autonomy, and inclinations to invest in distinct natal kindreds. These conflicts can get ugly, even lethal. We present the results of a pilot study conducted in Bangladesh which suggests that heightened mortality arising from mother-in-law/daughter-in-law conflict may be a two-way street, and we urge others to replicate and extend these analyses.

## Introduction

### The Good

Darwin’s theory of evolution by natural selection implies that the fundamental “interests” of any focal actor-the preferences, tastes, ambitions, and aversions that are characteristic of its species-will typically be such that the pursuit of those interests tends to promote the actor’s fitness (Darwin, 1859). Hamilton’s extension of Darwin’s theory further implies that the interests of two parties will tend to be harmonious in direct proportion to their degree of genetic relatedness (Hamilton, 1964). However, common ancestry is not the only source of shared fitness interests. Unrelated marriage partners often have a deep concern for one another’s well-being, and this, too, has shared fitness interests as its ultimate basis. Indeed, under certain conditions, unrelated mates may attain a level of common purpose that surpasses that of even the closest genetic relatives. Richard Alexander stated those conditions as follows:

“To the extent that 1) philandering is unlikely or too expensive to be profitable, and 2) the relatives of one or the other are not significantly more available for nepotistic diversions of resources, each member of the pair will profit from complete cooperation with the other to produce and rear their joint offspring.” (Alexander, 1987: 70)

A couple’s shared interest in their mutual progeny engenders common purpose that extends beyond the mated pair to their respective kindreds, and hence to various parties who are related affinally (by marriage) even if not genetically (by ancestry). The father of an infant and its maternal uncle (the mother’s brother), for example, will both gain fitness from the child’s eventual reproduction, so both men have reason to invest in the child’s future, and these brothers-in-law may therefore come to value the continued existence and well-being of one another as well. James Dow was apparently the first evolutionary anthropologist to see the implications. In a critical commentary on a Hamiltonian analysis of human cooperation, Dow (1984) argued that affines cooperate because of “the increased fitness caused by coancestral cooperative investment in common descendants” and that genetic relatedness is therefore inadequate as an index of shared fitness interests.

Austin Hughes (1988) was evidently unaware of Dow’s commentary when he, too, proposed that an alternative way to compute socially relevant degrees of kinship from genealogical data is on the basis of “relatedness to descendants rather than ancestors,” and in particular, on the basis of overlaps of relatedness to youngsters of high reproductive value. Hughes proceeded to demonstrate the utility of this “upside-down” approach to kinship by using it to explain some otherwise puzzling ethnographic data. In one particularly compelling example, he re-analyzed the genealogical data from a study of a Tennessee mountain community in which every resident was considered a member of one of four named “families”. Having found that patrilineal descent from the four ostensible male founders could not explain family membership, the ethnographer, Carlene Bryant (1981), had thrown up her hands and declared that the assignment of individuals to families followed no “rule”. However, Hughes (1988) neatly demonstrated that every individual in Bryant’s data set could be allocated to the correct nominal family on the basis of their degrees of relatedness to all the hitherto unmarried young people in the community.

In short, calling affines “kin” is more than a mere metaphor. When persons related by marriage have “fitness vehicles” in common, they become vehicles of one another’s fitness, too. If your daughter-in-law will bear and raise your son’s children, then she is a propagator of your genes no less than is he. In a recent theoretical paper entitled “Inclusive fitness for inlaws”, Dyble et al. (2018) proposed that this insight warrants a formal expansion of inclusive fitness theory to incorporate affines. Substituting a “coefficient of shared reproductive interests” [*s*] for Wright’s coefficient of genetic relatedness [*r*], Dyble et al. revised Hamilton’s famous inequality expressing the condition for altruism to be favored by selection from [*rb > c*] to [*rs > c*]. After working through some equations, examples, and complications (the most important being that “unlike genetic relatedness, shared reproductive interest is not usually symmetrical”), the theorists wrapped up their discussion as follows:

“Hamilton [2, p. 16] famously wrote that, on the basis of inclusive fitness theory, “we expect to find that no one is prepared to sacrifice his life for any single person but that everyone will sacrifice it when he can thereby save more than two brothers, or four half-brothers or eight first cousins”. According to our definition of the coefficient of shared reproductive interest, we might also add “or two daughters-in-law or eight cousin’s spouses”. (Dyble et al., 2018: 3)

So do people in fact view in-laws as members of their kin group, and behave accordingly? Burton-Chellew and Dunbar (2011) proposed to “test Hughes’s hypothesis” that affines are psychologically like genetic relatives and unlike unrelated friends by analyzing a social network data set in which respondents had both rated their emotional closeness to various individuals and reported how much time had elapsed since their last contact with them. The relationship between these variables was virtually identical for genetic kin and affinal kin, but quite different for unrelated friends, and from this and other analyses of contact that incorporated degrees of “relatedness” in which “a sibling’s spouse is treated as having the same nominal relatedness to ego as his or her sibling” (p. 742), Burton-Chellew and Dunbar concluded that Hughes was correct: “In effect, people treat affines as biological kin rather than as unrelated friends because... in-laws share with ego a common genetic interest in future generations” (p. 745).

A criticism of this analysis is that it treats social relations as if they were simply pairwise interactions with no larger social context. Burton-Chellew and Dunbar (2011) posit that “Actively contacting an alter or meeting up with them face-to-face involves time and commitment, and how often an ego contacts alters is thus a measure of his or her investment in that alter” (p. 745), but in reality, “egos” and “alters” frequently come together without either having “actively contacted” the other and this may be especially true of in-laws, as a result of “family” gatherings. If, for example, my sister and I exchange dinner invitations that include our spouses and I thus have the same frequency of contact with my brother-in-law as with my sister, it does not follow that I am “investing” similarly in them. Nevertheless, there is no question that people often *do* cooperate with affines and derive significant support from them (e.g., Cronk et al., 2019; Power and Ready, 2019).

Claude Lévi-Strauss (1949) famously maintained that marriage is a contract not between individuals but between kin groups, and moreover that it is fundamentally an exchange of women between patrilineages. Few anthropologists now accept that patrilineal descent reckoning is universal or primordial, but Lévi-Strauss’s insight remains relevant. Kin groups everywhere play a major role in determining who marries whom, and in specifying what entitlements and obligations the marriage will entail, both for the wedded couple and for their relatives. Marital negotiations are political, and the establishment or strengthening of advantageous affinal kinship ties is a primary agenda of the negotiators (e.g., Potter, 1934; Radcliffe-Brown and Forde, 1950; Schutte, 2020).

Shane Macfarlan and colleagues have shown that affinal relationships are crucial for building and maintaining coalitions of warriors in Amazonian hunter-horticulturalist societies in which raiding and lethal violence are common. In a paper entitled “Bands of brothers and in-laws...” Macfarlan et al. (2018) demonstrate that assembling a raiding party among the Waorani of Ecuador routinely exploits existing affinal links, and that nurturing relationships with potential affines by cooperating in multiple raids creates new marital opportunities both for the warriors and for their children. A similar logic prevails among the Yanomamö of Venezuela who collaborate in lethal acts of war mainly with men who are close in age to themselves and whose genealogical relationships are such as to make them appropriate marital exchange partners, with the result that co-killer dyads often marry one another’s sisters (Macfarlan et al., 2014).

Among contemporary hunter-gatherers, adult brothers and sisters often reside together, and the average adult individual actually has more marital relatives than genetic relatives in the band within which he or she currently resides (Hill et al., 2011). It is noteworthy that these affine-drenched social milieus are the very ones within which norms of equitable within-band sharing and an aversion to hierarchy commonly prevail (Boehm, 2009), in contrast to the unequal social arrangements in the patrilineal residence groups that are more characteristic of settled agriculturalists and pastoralists. It is also worth remarking that these egalitarian hunter-gatherer bands still provide our best available models of the social environments of human evolution.

## The Bad

Marriages create and cement alliances, then, and affines can be assets and allies. But something is missing from the cozy picture of affinal harmony that we have painted thus far. There is a cross-culturally ubiquitous characterization of in-law relationships that is very different, namely that they are tense at best, and toxic at worst.

In-laws and their “meddling” rank high among the sources of conflict in contemporary marriages, often higher than such potential flash points as financial issues or mismatched values (Messinger, 1976; Silverstein, 1990; Bryant et al., 2001; Ward and Lin, 2020). Moreover, although the birth of a grandchild creates an overlap of fitness interests among all parties, this overlap doesn’t necessarily diminish tensions. In a Finnish study, Danielsbacka et al. (2018) found that young adults reported relatively little conflict with a partner’s parents before having children, but that such conflicts increased after the birth of a first child, and explain that “The shared reproductive interest that is created through a grandchild among kin lineages provides new reasons for grandparents to influence and interfere in the lives of other family members” (p. 79). In a Japanese study, Honjo et al. (2018) found that co-residing with parents-in-law was associated with significantly elevated rates of post-partum depression (PPD), net of various potential confounds, whereas dwelling with the new mother’s own parents was associated with significantly reduced rates.

Much, perhaps most, of the serious conflict between husbands and wives derives from the former’s efforts to thwart the latter’s pursuit of their own interests (Wilson and Daly, 1992), and affines are often drawn in. A mother-in-law, for example, sometimes sees her role vis à vis her daughter-in-law primarily as one of mate-guarding on behalf of her son (Voland and Beise, 2005). Abuse of young wives by their mothers-in-law has been a focus of special concern because of the serious harms that it often entails (Voland and Beise, 2005; Raj et al., 2011; Venugopal, 2014; Devnath, 2021), but a despicable mother-in-law is a reliable stock character in the lamentations of husbands, too (Try Googling “mother-in-law jokes,” and you will find many thousands of examples.)

Affinal relationships, including marriage itself, are more fragile than those among genetic relatives for reasons that have been elegantly expressed by David Haig:

“The love of a child is more robust to bad behavior by the child than is love of a spouse to bad behavior by the spouse. The sharing of genes by descent is a brute fact that is unchanged by changes in the personal relations of kin, but spousal fitnesses are decoupled when either partner pursues other reproductive opportunities.” (Haig, 2011: 10881).

Our only quarrel with Haig’s pithy summary is that his phrase “other reproductive opportunities” can be interpreted as narrowing the analytic focus from the full range of activities promoting inclusive fitness to personal reproduction. Evolutionists often privilege “paternity uncertainty” and the risk of “cuckoldry” as the primary or sole ultimate source of the tension between female autonomy and male control, but the issues are broader. For one thing, even in a species where males invest nothing in their putative offspring and therefore cannot literally be cuckolded, they are nevertheless motivated to monopolize sexual contact with their mates (e.g., Lott, 1981); similarly, when men take exception to their wives leaving them, their concern is not that they might misdirect their paternal investments to the wrong children. Moreover, the ultimate basis of marital conflict resides not only in personal reproduction and direct fitness, but in the entire array of fitness-promoting activities. Alexander (1987), whom we quoted at the outset, saw this clearly when he identified the second main threat to marital solidarity as “nepotistic diversions of resources” to the couple’s distinct kindreds.

The issue here closely parallels that which Perry and Daly (2017) explicated with respect to evolutionary explanations of preferential investment in “uterine” grandchildren (the children of daughters) over “agnatic” grandchildren (the children of sons). Even if paternity were certain, selection would continue to favor this preference because an investment in a grandchild is also an investment in its mother, raising her capacity to pursue her fitness interests in other ways, and one’s own inclusive fitness is better served by raising the nepotistic potential of one’s daughter than by raising the nepotistic potential of one’s daughter-in-law.

It follows that Burton-Chellew and Dunbar (2011) over-simplified a complex social reality when they elected to treat siblings and siblings-in-law as “having the same nominal relatedness to ego” (p. 742), and that Dyble et al. (2018) made the same error when they modeled the “shared reproductive interest” that one has with one’s daughter and one’s daughter-in-law as identical (*s* = 0.5 in both cases). To their credit, Dyble et al. acknowledge that this is overly simplistic in their Supplementary Materials, where they offer an expanded model that includes a paternity uncertainty parameter. But that remedy addresses only the first of Alexander’s threats to marital solidarity, while ignoring the second, and it addresses even the first incompletely. One reason why your daughter-in-law does *not* have the same expected value for your inclusive fitness as your daughter is that her reproductive career will not necessarily be monogamous for reasons of widowhood, divorce, and remarriage in addition to paternity uncertainty (Voland and Beise, 2005). But another reason why the daughter-in-law is of lesser value to you is that the people whom *she* values and might invest in are less closely related to you, on average, than the people whom your daughter values and might invest in (Perry and Daly, 2017). Every marriage counselor has heard husbands complain that their wives spend “too much” time or attention on members of their natal families.

In short, when marriage partners reproduce, the resultant overlap of their fitness interests enhances their value in each other’s eyes, and in the eyes of one another’s relatives, too. But couples also experience a range of conflicts that seem qualitatively distinct-conflicts over commitment, shirking, interactions with their respective families, and so forth-but all derive from the fact that each partner’s interests would be best served if the other devoted his or her entire reproductive and nepotistic effort budget to their mutual progeny. And inlaw conflicts shadow these spousal conflicts.

## The Ugly

The stakes are often high in conflicts among persons related by marriage, and it should be no surprise that they can become vicious, even lethal.

The majority of homicides in which the victim and the killer were “related” adults are spouse-killings (Daly and Wilson, 1988). The precipitating circumstances usually involve either (suspected) infidelity or the woman’s attempting to leave the man, and other affinal relatives may be “collateral damage” in such cases. A recurrent variety of homicide that is virtually confined to affines is that in which the victim is slain for “harboring” the killer’s estranged wife. Typical victims in these murders are the killer’s father-in-law or brother-in-law, but female in-laws may be killed too. Moreover, in samples of homicides from diverse societies, affines other than spouses comprise a proportion of all victim-killer relationships that is surprisingly high when one considers their relatively low rates of contact and engagement. A sample of 100 adjudicated homicides among the Bhil (Varma, 1978), a patrilineal “tribal” group in India, provides an example: seven men were slain by brothers (including one paternal half brother who was erroneously labeled a “step-brother”) and an identical number by brother-in-laws, which is remarkable because the latter typically live far apart and rarely meet, whereas brothers see each other every day and are engaged in chronic disputes over family property. For other such examples, see chapter 2 of Daly and Wilson (1988).

The definition of an affine is a “relative by marriage,” and although stepchildren are seldom mentioned in discussions of affinal kin, that is clearly what they are. Like other affinal relationships, those between stepparents and their stepchildren are often cooperative, supportive, and even affectionate, but they are also risky and fraught. Most stepchildren are well cared for, but many studies in many lands have demonstrated that they receive lesser average investments of various sorts than children living with both their genetic parents, and that stepchildren are massively over-represented as victims of physical abuse, sexual exploitation, and fatal battering (review by Daly and Wilson, 2008).

The presence of stepchildren is also an important source of marital conflict (Daly and Wilson, 1996), and a major predictor of uxoricide, the killing of one’s wife (Daly et al.,1997; Campbell et al., 2003). The news organization *Stuff* maintains a public archive of all homicides known to have taken place in New Zealand since 2004 (Fyers and Ensor, 2021). Between 2004 and 2018, 117 women were reportedly slain by their husbands, and our examination of the news coverage of the killings and the trials reveals that no fewer than 49 of the victims (42%) were the mothers of children sired by previous partners. Since the coverage of individual cases was not always fulsome and one in five wife-killers avoided a trial by committing suicide, that 42% is surely an underestimate.

The most notorious category of homicidal violence by in-laws is that of so-called “dowry deaths” (Babu and Babu, 2011) or “bride burnings” (Kaur and Byard, 2020) in the Indian subcontinent. The former term signals that these killings occur in the context of demands for dowry payments that the bride’s family cannot or will not meet, and the latter that the immediate cause of death is most often a “kitchen fire”. In India alone, between 6,000 and 9,000 young wives die each year in what are officially recognized as dowry-related crimes (Government of India, 2021). In reality, the toll is surely much higher, since the great majority of such deaths are officially recorded as accidents or suicides rather than as homicides (e.g. Khartade et al., 2014; Verma et al., 2015).

### Deadly Daughters-in-Law? A Pilot Study

In April and May, 2014, the authors were located at the *International Centre for Diarrhoeal Disease Research, Bangladesh* (icddr,b) in Matlab, Bangladesh, where one of us (GP) was conducting interview research on alloparental care and assistance (Perry, 2017a; Perry, 2017b; Perry, 2021). In the Bengali society of Bangladesh and adjacent northeast India, it is normative that a young bride should move into her husband’s natal family home at marriage, and abuse and exploitation of women by their mothers-in-law is a widely acknowledged problem (e.g., Aziz, 1985; Rohner and Chaki-Sirkar, 1988; Bates et al., 2004). More specifically, it is clearly an issue in Matlab (Ahmed et al., 2004).

Many interviewed women complained of mistreatment by their mothers-in-law, and almost all declared a preference for returning to their natal homes to give birth (Perry, 2017b). Meanwhile, our primary news source, the English language *Dhaka Daily Star*, carried frequent stories about violence against daughters-in-law, some of it lethal. In Bangladesh, as in India, “suicides” of women living with their in-laws are often thinly disguised murders (Ahmed et al., 2004; Bagley et al., 2017; Devnath, 2021). We therefore wondered whether and to what extent living with one’s mother-in-law might be associated with excess mortality of Matlab women. The data that we were able to amass indicate that such an excess indeed exists, but were too few to provide a good estimate of its magnitude, and to our surprise, what was even clearer was excess mortality among the mothers-in-law themselves. Although the study is preliminary, this result warrants a brief report.

The icddr,b has long maintained a program of population and health monitoring in the predominantly rural area of Matlab, where most people reside in family compounds (*baris*) containing one or more households, separated from one another by seasonally flooded rice paddies. Since 1966, the icddr,b’s Health and Demographic Surveillance System (HDSS), 2015 has frequently censused a local population of about 230,000 people, recording all births, deaths, marriages, divorces, in- and out-migrations, and changes of residence, usually bi-monthly. Rahman (1999) used these data up to 1994 to show that having a spouse significantly reduced mortality of elderly men, but not women, whereas having sons and (especially) living brothers reduced the mortality of women; he did not look for possible in-law effects.

The HDSS data were available to us not as a computer file, but in a set of more than 800 bound volumes, which detailed household compositions as of December 2012. To supplement GP's interview data, MD extracted information from these volumes on the residential household compositions of a 10% random sample (N = 8389) of Matlab women 15 years of age or older, recording each focal woman’s age and current marital status; the total number of persons in the household; how many of those present were her children, her grandchildren, and her daughters-in-law; and the presence or absence of her husband, her mother, and her mother-in-law. Bimonthly updates entered by hand indicated that 71 of these women (0.85%) had died in the ensuing 14 months (January 2013 through February 2014), and MD also recorded that as a binary variable (survived/died).

The age distribution of women in our 10% sample was close to that reported for the local population as a whole (icddr,b 2015), providing some assurance that the sample was reasonably representative. Among women 15–24 years of age, 30.0% dwelt with the mother-in-law, which represents 61% of those currently married, as 50% of this youngest age group had yet to marry. Marriage is almost universal, however, and in the 25–34 year-old group, only 4.3% were still never-married, but just 34% of the married women dwelt with mothers-in-law, mainly because over time, couples moved into homes of their own, often but not always within the same patrilineal bari, and women whose husbands were migrant laborers frequently resided with their own mothers (Perry, 2017a). By age 35–44, only 18% of currently married women lived with their mothers-in-law, and by 45–54, that percentage was just 8.5%.

**FIGURE 1:**
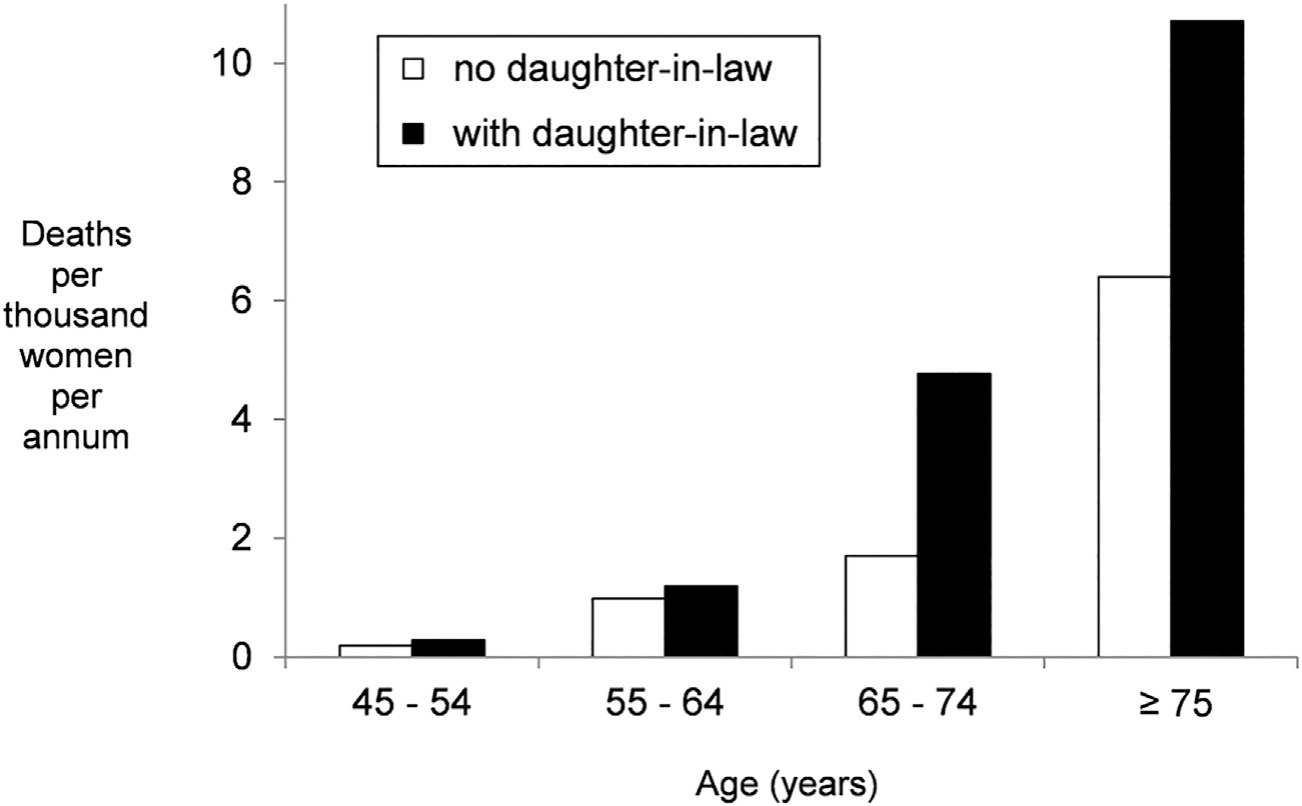
Annualized mortality rates of women with and without co-residing daughters-in-law, based on the 10% sample.

Table 1 shows the numbers of deaths that occurred in the ensuing 14 months, as well as the age-specific death rates in our sample and in the entire adult female population of Matlab in 2013 (icddr,b 2015). Sample deaths are about as numerous as expected, but because only 8 of the 6,722 women under 55 years of age died, and women in older age groups hardly ever resided with mothers-in-law, a robust assessment of the effect of coresiding mothers-in-law on mortality was precluded. In older age groups with higher mortality rates, however, many women dwelt with daughter-in-laws, and Figure 1 shows that age-specific mortality of women residing with daughters-in-law was elevated in comparison to other same-age women.

**TABLE 1:** Sample size, living arrangements, and death rates, by age group, in the 10% sample of Matlab women.

Age group	Sample N	Percentage (N) living with mother-in-law	Percentage (N) living with daughter(s)-in-law	Observed N of deaths (Jan 2013 thru Feb 2014)	Sample death rate per 1,000 per annum	Population death rate per 1,000 per annum, 2013
15–24	2,021	29.8 (603)	0	2	0.9	1.1
25–34	1,768	31.7 (560)	0	2	1.0	0.9
35–44	1,501	17.6 (264)	2.3 (35)	1	0.6	0.8
45–54	1,432	7.1 (102)	24.7 (353)	3	1.8	3.6
55–64	829	2.1 (17)	50.5 (419)	9	9.3	11.0
65–74	579	0.2 (1)	65.1 (377)	25	37.2	29.3
>74	259	0	74.1 (192)	29	96.4	97.0

The local population-at-large death rates are derived from data in Tables 3.3 and 4.1 of icddr,b (2015).

Table 2 presents the results of two logistic regressions assessing the apparent effects of various categories of coresiding relatives on a woman’s odds of dying in the 14 months after the December 2012 census. Separate regressions were run for two age groups (under 45 vs. 45 and older) because the former group rarely had a coresiding daughter-in-law and the latter rarely had a coresiding mother-in-law (Table 1). The effects of age were controlled by including a predictor variable set equal to the age-specific death rates of Bangladeshi women in 2010 according to the World Health Organization (2021); this variable had no predictive value for women under 45, for whom mortality rates are a relatively flat function of age, but was a highly significant predictor for the older group, for whom mortality rates rise steeply with age.

**TABLE 2:** Results of two logistic regressions assessing the apparent impacts of other household members on the mortality of women in two age groups, during a 14 month period beginning on January 1, 2012.

a. Women under 45 years of age (*n* = 5,290)			
Predictor	Odds ratio	95% C.I.	*ρ*
Expected mortality for age	—	—	0.792
N of persons	0.59	0.29–1.20	0.147
Husband	0.56	0.81–3.78	0.548
Own child(ren)	2.02	0.20–20.51	0.553
Mother-in-law	7.68	1.07–55.39	0.043
**b. Women 45 years of age and older (*n* = 3,099)**			
**Predictor**	**Odds ratio**	**95% C.I.**	***ρ***
Expected mortality for age	—	—	0.000
N of persons	0.95	0.82–1.10	0.506
Husband	0.54	0.29–1.02	0.056
Own child(ren)	0.88	0.47–1.65	0.691
Daughter(s)-in-law	3.59	1.81–7.10	0.000

As anticipated, residing with one’s mother-in-law was associated with an elevated risk of mortality for the younger women. The estimated odds of dying are over 7-fold higher than for same-age women, but the significance level is marginal and the confidence interval is large. More precise estimation of the mother-in-law’s impact would require a larger data set. For the older women, living with a daughter-in-law was also associated with an elevated risk of mortality, and in this case, although the Odds Ratio is smaller, the effect is highly significant. In both age groups, residing with a husband appears to be protective (Odds Ratio < 1), but in neither case is the effect statistically significant.

A potentially important missing control variable in Table 2 is socioeconomic status. It is conceivable that the apparent in-law effects result from a confound whereby the households in which mothers-in-law and daughters-in-law co-resided were relatively poor. Data from GP’s interviewees, however, suggest that any such confounding is likely to be slight, because the means and distributions of household incomes (adjusted for household size) were similar for those who did and those who did not reside with the mother-in-law (Perry, 2021).

If, as we suspect, the daughter-in-law “effect” in Table 2 is genuine, it need not reflect acts of overt violence or malevolence. After years of domination and perhaps even abuse by her mother-in-law, a mature woman to whom a growing share of power has shifted may simply be relatively inattentive to her elderly mother-in-law’s needs. Mortality due to cholera, for example, has fallen dramatically in the Matlab area thanks to the icddr,b staff and hospital, but residents still contract the disease at high rates and rapid response is essential.

Elevated mortality when in-laws co-reside may also be mediated by the effects of chronic conflict and stress. In a Japanese study, women (but not men) had greatly elevated rates of coronary heart disease, and of death therefrom, in “three-generation” households (Ikeda et al., 2009); unfortunately, mother and mother-in-law effects are indistinguishable in the data base for this study (I. Kawachi, personal communication), but Japanese couples live with the husband’s parents more often than with the wife’s, and other research suggests that the negative impact is likely to be a mother-in-law effect.

Nishi et al. (2010) assessed the survival of 129 elderly women in a mid-size Japanese city over 51 months, and found that those whose primary care-giver was a daughter-in-law (n = 48) had the highest mortality, while those cared for by a spouse had the lowest (n = 19). Despite the very small numbers, this difference was statistically significant, net of age and other controls, but mortality levels when cared for by a biological daughter (n = 24), another relative (n = 20), or no-one (i.e., living alone, n = 18) were all intermediate between the daughter-in-law and spouse groups and significantly different from neither. As far as we know, this is the only prior study suggesting that daughters-in-law may raise the mortality of their mothers-in-law.

The analyses presented here could, in principle, be conducted with data for the entire Matlab population, rather than just a 10% sample, and over many years. This would afford greater statistical power for determining whether mothers-in-law and daughters-in-law indeed exert reciprocal effects on one another’s survival. Unfortunately, we are not in a position to carry out those more extensive analyses, and we encourage others to pursue these questions in this and in other populations.

## Conclusion

Dow (1984) and Hughes (1988) proposed that affinal “kinship” is no mere metaphor. The commonality of interest among persons related by marriage derives from the same ultimate source as the commonality of interest among persons related by blood. In both cases, the protagonists are “related” by virtue of the fact that they can expect to derive fitness from the same particular reproductive events.

We show, however, that recent theoretical arguments that treat daughters and daughters-in-law (for example) as equivalent contributors to ego’s fitness go too far. “Parallel” pairs of genetic and affinal relationships such as these are importantly different, both quantitatively and qualitatively. Any child of my daughter will be my grandchild, but that is not necessarily true of my daughter-in-law, and even if the latter were to reproduce only with my son, she would retain an interest in natal relatives who are of no relevance to me. The “brute fact” of genetic relatedness (Haig, 2011) favors forgiveness and reconciliation among blood kin, even after betrayals, but a daughter-in-law, unlike a daughter, is replaceable (Voland and Beise, 2005; Mace, 2013). Bride-burnings are committed by mothers-in-law, not by mothers. The oppressive mistreatment of young women by their mothers-in-law, especially in the Indian subcontinent, has been much remarked upon, but we show, in addition, that the destructive effects of this relationship can be a two-way street.

According to Leonetti et al. (2007) “We can speak of “in-law conflict” as an extension of sexual conflict, with parents on both sides joining the fray. Cooperation may also be part of these relationships when the interests of both sides are enhanced. This game, of course, becomes vastly more complicated than the simple struggle between the sexes but is likely to be ancient and of critical importance to human reproductive success.” We concur.
